# Impact of Biotic and Abiotic Stressors on Managed and Feral Bees

**DOI:** 10.3390/insects10080233

**Published:** 2019-08-01

**Authors:** Joseph Belsky, Neelendra K. Joshi

**Affiliations:** Department of Entomology, University of Arkansas, 319 Agricultural Building, Fayetteville, AR 72701, USA

**Keywords:** bee declines, floral diversity, pesticides, parasites, competition, climate change

## Abstract

Large-scale declines in bee abundance and species richness over the last decade have sounded an alarm, given the crucial pollination services that bees provide. Population dips have specifically been noted for both managed and feral bee species. The simultaneous increased cultivation of bee-dependent agricultural crops has given rise to additional concern. As a result, there has been a surge in scientific research investigating the potential stressors impacting bees. A group of environmental and anthropogenic stressors negatively impacting bees has been isolated. Habitat destruction has diminished the availability of bee floral resources and nest habitats, while massive monoculture plantings have limited bee access to a variety of pollens and nectars. The rapid spread and increased resistance buildup of various bee parasites, pathogens, and pests to current control methods are implicated in deteriorating bee health. Similarly, many pesticides that are widely applied on agricultural crops and within beehives are toxic to bees. The global distribution of honey bee colonies (including queens with attendant bees) and bumble bee colonies from crop to crop for pollination events has been linked with increased pathogen stress and increased competition with native bee species for limited resources. Climatic alterations have disrupted synchronous bee emergence with flower blooming and reduced the availability of diverse floral resources, leading to bee physiological adaptations. Interactions amongst multiple stressors have created colossal maladies hitting bees at one time, and in some cases delivering additive impacts. Initiatives including the development of wild flower plantings and assessment of pesticide toxicity to bees have been undertaken in efforts to ameliorate current bee declines. In this review, recent findings regarding the impact of these stressors on bees and strategies for mitigating them are discussed.

## 1. Introduction

Bees provide integral pollination services enabling the fruiting and reproduction of both agricultural food and fiber crops [1] and wild plant species [2]. Honey bees (*Apis* species with primary emphasis on *Apis mellifera*) are domesticated for their pollination services and production of a variety of hive products, including honey, throughout most of the world. They are generally considered to be the most valuable pollinators of monoculture crops given their perennial biology, large workforce of foragers, ecology as non-specific pollinators, widescale availability, ease of transportation, and economic affordability [1,3]. Bumble bees (*Bombus* species) are considered to be a foundation for natural ecosystems given their generalist pollinator ecology and the complexity of the flowering plants that they forage in diverse habitats, particularly in mountainous regions where they are most abundant [4,5]. They are unique in their ability to pollinate flowers requiring high frequency sonication [6] and capability of pollinating in cooler regions at wider daily timescales. Solitary bees comprise the majority of native bee species, and the high value of their pollination services has been recently elucidated [7,8]. A handful of solitary bees are commercially managed for crop pollination services; for example, mason bees, (*Osmia* species) are used to pollinate orchard fruits, while alfalfa leafcutter bees (*Megachile rotundata*) are used to pollinate alfalfa (*Medicago sativa*) and other forage crops [9].

Several events over the last decade have marked a recent large-scale decline in bee abundance and species richness that has caused great concern. Beginning with Colony Collapse Disorder (CCD) impacting honey bees in the United States, commercial beekeepers have experienced approximately 30% annual losses of overwintering colonies, marked by a high of 45% in 2012–2013 [10]. Although various stressors have been identified in contributing to CCD, no single factor has been isolated [11]. Globally, pollinating animals are crucially important, given their substantial contribution to crop yield and resulting output. For 2009 (but inflated to 2015 USD), an animal-mediated addition of US$235–577 billion was estimated [12]. These benefits are not evenly distributed, with regions growing the most obligate animal-pollinated crops receiving the highest impact [13]. Additionally, insect-pollinated crops provide crucial micronutrients and dietary fiber for human health [1,14]. A prime example is rampant Vitamin A deficiency in many world regions, which in part results from shortages of obligate animal pollinated crops that provide up to 70% of Vitamin A; while self-pollinated crops providing Vitamin A can increase yield by 43% when pollinated by animals [14]. Finally, pollinators are crucial for the proper functioning of ecosystems and food webs, and their decline would have serious implications for many ecological niches, particularly in tropical zones where their services are of highest dependence [15,16]. Populations of bumble bees and native bees (including many solitary bee species) have also steadily decreased over the past 50 years, with several species having major downfalls and a few becoming endangered or extinct [17]. Declines in wild bees and other pollinating species have been documented in preliminary reports such as [18,19]. Addressing disagreement regarding these declines, a recent study examined the data and proposed that a combination of stressors have impacted both *Apis* and non-*Apis* bees [17]. This paper has been subsequently criticized by papers stating that pollinator declines mainly concern managed honey bees and bumble bees in North America and Europe [20]. However, a range of studies providing a broad evidence base over the last decade have shown significant reductions in the abundance and diversity of bumble bees and solitary bees in different world regions [21,22,23,24,25]. Additionally, the increased understanding of solitary bee vitality in pollinating agricultural crops and wild plants within their native habitats has been recently elucidated through a myriad of bee ecological studies [26,27,28,29]. At the same time, demand for bee pollination services has substantially increased, given the surge in the cultivation of obligatory-bee pollinated crops in both the developed and developing world [30]. Therefore, bee researchers have become substantially interested in understanding the stressors impacting solitary bees (with particular emphasis on a handful of commercially domesticated species). In our opinion, the highest priority in future solitary bee research programs are elucidating a) how interactions of biotic and abiotic stressors exacerbate solitary bee declines, and b) a molecular-level determination of stressor impacts. While we have not reached a breaking point where crop cultivation has surpassed the quantity of pollinating bees, data indicate yield reductions at localized levels resulting from insufficient pollination [17].

These substantial bee declines have given rise to a flurry of research investigating potential bee stressors [2,31,32]. This review explores recent advances in scientific research that builds and expands upon current knowledge in bee stressors, as part of a continuous effort to protect managed and feral pollinating insects. For example, a recent study demonstrating that on a global level, only a small subset of common wild bee species visit crop production systems justifies the need for more concerted bee conservation research that expands beyond protecting bees on the premise of their pollination services [8]. This new paradigm of research should address the intricate stressors that bees are exposed to in their ecological environments, and how these combined stressors exacerbate the current decline of bees worldwide.

From a habitat perspective, future work may investigate the impact of biotic and abiotic stressors on a certain bee taxon in regards to a specific landscape composition. Findings that show that a loss of floral diversity and abundance results in decreased food availability and thus is a direct impact of habitat manipulation on bees begin to address questions related to this topic [33]. This review plus others demonstrate the highly variable pollinator responses to land change that differ at local versus surrounding gradient scales [34]. This creates an opportunity for additional studies to investigate the effect of indirect factors such as landscape context, disturbance, and invasive species on specific pollinator taxa when floral resources are maintained constant. Also, most landscape context studies that result in negative impacts on pollinators have made extreme landscape manipulations [34]. Future work can re-examine these questions by making more subtle changes that are more representative of agro-ecosystems. Several recent efforts have been made to tease apart the specific nutritional needs of bees, and specifically non-*Apis* species. For example, it has been proposed that ecosystem restoration for pollinators should not only consider the unique pollen and nectar requirements necessary for balanced diets of adults and larvae of individual species, but also the provisioning of adequate floral resources throughout the spectra of a day and temporal season [35,36]. This approach accounts for differences in species-specific foraging behaviors and addresses both generalist and specialist pollinators, thereby enabling us to create target plant species for floral restorations that are tailored to specific regions and habitats. Similarly, researchers argue that future studies on how floral resource availability influences bee population dynamics should key in on a) intricate nutrients and their direct impact on bee health, b) synergisms between bee nutrition and other environmental stressors such as pesticides and pathogens, and c) how the removal of one plant species from an ecosystem alters bee–flower preferences and results in the decline of certain bee species [37]. A focal question of future work might explore the synergistic effect of stressors on bee learning and memory.

Bees are naturally afflicted by a number of parasites, pathogens, and pests; honey bees are the species for which we understand these maladies best. Current studies have linked *Varroa* mites as the vector of a large number of honey bee viruses [38,39]. Molecular level analysis has confirmed this symbiosis [40], while other works have isolated the detrimental effects of combined interactions between viruses and pesticides on honey bee health [41]. Similarly, new approaches using molecular biology have been demonstrated for understanding how other honey bee maladies such as *Paenibacilius larvae* [42] and *Nosema* microsporidia [43] are vectored, and outlining corresponding appropriate management tactics. More recent literature has explored the spread of bumble bee and solitary bee parasites on a global level [44,45,46,47]. These initial findings have inspired the use of molecular tools to isolate historical time periods correlated with rapid levels of bumble bee parasite infestation [48] and pattern mapping for parasite spread among bee species [49]. A further step has investigated pathogen spillover from infected commercial bumble bee colonies to wild species [50], and their possible role in wild species declines has been proposed [51]. We encourage the future direction of bee disease research to build upon this molecular route, with particular concern for non-*Apis* species. Also, more detailed mapping of pathogen spillover from managed pollinators that encompasses spread to solitary bee species should be emphasized. Finally, it is essential to build upon the recent findings that extrapolate synergisms between disease and other environmental stressors in honey bees [41,52] and to investigate if these synergisms also exist for non-*Apis* species.

Pollinators may directly encounter pesticides through a variety of different routes including oral and contact exposure with contaminated pollen, nectar, and water, bodily contact exposure with spray applications, and contact with residues on plant leaves, stems, and soil [53,54]. Additionally, herbicides can impose indirect stress on pollinators by contacting flowering plants, providing nectar and pollen food resources and subsequently resulting in their loss or elimination [2]. Most of the current literature regarding pesticide impacts on bees has focused on determining toxicity following application of a field realistic dose of a specific chemistry to a single species (or small group of species) in either a laboratory or field setting [55,56]. However, more recent studies have approached this topic from the angle of how the unique biology and ecology of a handful of individual non-*Apis* species leads these bees to encounter pesticides in a myriad of settings [57,58,59,60]. Moreover, a few studies have suggested that bee exposure to pesticides in combination with other environmental stressors may impose synergisms that together result in currently observed bee declines [16,17]. Our review explores the findings of these studies and makes a case for pollinator–pesticide studies to take a new direction quantifying overall risk assessment (as opposed to simply toxicity) from pesticide exposure in a variety of mediums relevant to *Apis* and non-*Apis* species environmental interactions. In this new approach, we also emphasize viewing the synergized stress imposed on bees from exposure to pesticides and other stressors. Finally, we look at studies exploring the molecular basis for honey bee pesticide detoxification [61,62] and suggest that additional work should build upon these, particularly focusing on non-*Apis* species.

The series of new studies described above bring forth exciting findings that consistently outline a group of bee stressors shown to individually and specifically in combination interact to negatively impact bees and hence drive their population declines. (1) Habitat destruction and fragmentation has decreased the availability of bee floral resources and nesting areas [18,63]. (2) Monoculture plantings have diminished the diversity of floral plant species within the radius of bee foraging distances [64,65]. (3) Parasites, pathogens, and pests that naturally afflict bees and vector bee diseases have rapidly spread and developed resistance to control tactics [66,67]. (4) Dramatic increases in (a) pesticide applications in agricultural systems to control insect pests [68,69], (b) honey bee hives to control bee pests and diseases [70,71] as a result of their resistance development, and (c) pesticide residues in nesting materials used by non-*Apis* species [72,73] simultaneously expose bees to these chemistries. (5) Human-mediated bee movements have been linked to inducing colony stress, introducing foreign parasites, and increasing resource competition for native bees [74,75,76]. (6) Researchers have proposed increased competition amongst bees within the same nest or ecological niche for limited resources as an additional driver of bee declines [77,78,79]. (7) Climatic alterations have disrupted synchronous bee emergence with flower blooming and reduced the availability of diverse floral resources [80,81]. (8) Interactions of multiple stressors impacting bees at the same time are thought to create cocktail-like effects that further intensify their sharp population declines [41,82,83,84]. (9) Several initiatives to aid bees have been established. For example, a variety of government and non-profit supported programs to develop wildflower plantings and pollinator gardens while simultaneously raising community awareness and engagement exist [85,86]. Efforts to minimize pesticide use, apply pesticides in manners less likely to affect bees, and increase the use of less toxic chemicals such as those derived from biological sources have also been undertaken [87,88].

Current research knowledge on stressors impacting bees has largely focused on Western honey bees (*A. mellifera*). Beginning with mass colony die-offs that are collectively referred to as CCD, honey bee health has been brought to the forefront of media, governmental, and environmental conservation efforts, when in reality, the primary concern of honey bees is for agricultural crop pollination and honey production [89]. A saturated focus on *A. mellifera* has created a lack of awareness of species diversity within the *Apis* genus, which contains eight extant species beyond *A. mellifera* in Asia [90]. However, bee scientists ranging from academia to government to industry, and organizations and environmental initiatives alike have recently recognized that pollinator conservation efforts must encompass the diversity of bee species, and that in some cases, the presence of honey bees in ecosystems may even negatively impact non-*Apis* bees [54,91]. In this context, it is presumed that complications in assessing the intricate roles of wild bees and the flowers that they pollinate in specific microclimates on a global scale has in part contributed to the shortage of data on their health and population status [54]; an excessive focus on the demise of managed honey bees may in part have clouded and dismissed these efforts. Nonetheless, the biotic and abiotic stressors impacting honey bees can be viewed as a harbinger to the likely declines of non-*Apis* bees (and particularly solitary species). On the other hand, efforts to aid honey bees such as minimizing pesticide application during crop bloom will likely also benefit non-*Apis* species that are present in the same agro-ecosystem [28,29]. To this regard, a thorough assessment of the precise need for managed honey bee colonies in crop pollination that (a) does not impact native bee species within the same ecological community, (b) accounts for non-*Apis* species pollination services (which may be up to 50% [92]), and (c) addresses periods when no mass flowering crops are in bloom to minimize honey bee competition with native bee species for wild plant pollination is necessitated [89].

In this review, we emphasize the importance of viewing bee declines through a complete lens that expands beyond traditional approaches of quantifying pesticide toxicity to a specific species and focusing on honey bees. By exploring the impacts of stressors on a group of commercialized solitary species, we set a platform for assessing the plight of the bees from a perspective that is ecologically wholesome yet still feasible for agricultural production and conservation goals. Our approach identifies and analyzes the recent literature on a group of biotic and abiotic stressors impacting the health, fecundity, distribution, abundance, and diversity of bees across different systems and world regions. In this context, we explore stress brought upon bees from common sources such as habitat manipulation, diseases, and pesticides, as well uncommon sources such as anthropogenic effects and climate change. Societal efforts to conserve bees and promote awareness within communities at large are discussed. However, our main emphasis is on the examination of potential synergisms arising from bee exposure to multiple stressors within their habitat, building upon the ideas put forth by other researchers [13,16,17]. Our goal is to contribute toward a transformational time in the science of bee health that fully encompasses the entire spectrum of species, the complexity of their interactions with their respective environments, and the potential multitude and impact of the stressors imposed upon them.

## 2. Habitat Destruction and Fragmentation

Disrupted landscapes lacking the diverse floral resources that are necessary for adequate bee nutrition within close proximity to where bees forage can substantially impair survival [93,94,95]. Obtaining proper nutrition during forage is essential for (1) adult bees to meet their food maintenance requirements and (2) the ability of adult female bees to provision their nests with appropriate food resources ensuring healthy offspring development. A major driver of the impact of habitat manipulation on bees is its key influence on the overall reduction of available floral nutrient resources. For example, an increased semi-natural habitat in agricultural landscapes was positively correlated with an increased abundance of cavity nesting bee species and growth rates of experimental *B. terrestris* colonies, but not the species richness of wild bees collected in pan traps [96]. Comparing bee visitation to cover crops, scientists have similarly found differences including phacelia (*Phacelia* species) mostly attracting honey bees and bumble bees, and solitary bees mainly visiting cultivated sunflower and wildflower mixes [97]. Hence, while floral diversity is important for providing bees with access to adequate food resources, there are trade-offs between serving common generalist species and more rare specialist species [8]. At a more specific level, recent findings demonstrate that in *Tetragonula carbonaria*, the diversity of pollen and resin species collected increases when provided access to a larger diversity of plant species, and the colony fitness and growth rate increase when surrounded by more florally diverse environments by measure of increased resource intake and food stores [98,99]. In honey bees, a decline in pollen harvest between rapeseed (*Brassica napus*) and sunflower, (*Helianthus annuus*) blossom was negatively related to colony brood output and subsequent colony adult population, as well as colony *Varroa* mite load [100]. A severe pollen decline between mass flowering events in intensive agriculture systems was found to reduce seasonal and overwinter survival by 50%. Therefore, the effect of food resource availability is directly related to outcomes in bee health, fitness, and overall survival. However, floral resource diversity is not the main factor of habitat manipulation, given growing evidence of the vitality of certain key plant species for proper bee nutrition. In a separate study examining stored pollen in beehives (bee bread), protein content was positively correlated with dandelion (*Taraxacum* species) pollen, and negatively correlated with cherry (*Prunus* species) pollen [101]. Similarly, in conducting a stoichiometric analysis of the elements that are necessary for honey bee growth, it was concluded that pollen lacking Na, S, Cu, P, and K, and possibly Zn and N may impart growth limitations that are different for each caste and sex [102]. Since concentrations of these essential elements reveal high floral taxonomic diversity, these authors recommend designing floral provisions around apiaries that contain (a) high plant species richness and (b) plant species that have stoichiometrically rich pollen, such as clover (*Trifolium* species). In non-*Apis* bees, it was shown that *O. bicornis* offspring survival, health, and female production are highly linked to the species on which pollen larvae feed, with the best results occurring for larvae provisioned with *B. napus* pollen [103]. Therefore, the pollen nutritional content of one plant species may influence that of other plant species, and bees overcome nutrient scarcity in poor resources by combining the pollens of multiple plant species. Building upon these findings, the results of another study demonstrate that proper *O. bicornis* growth and development necessitates sufficient quantities of K, Na, and N in pollen, while proper cocoon development may depend upon sufficient quantities of P, Mg, K, Na, Zn, Ca, and N in pollen [104]. In agreement with previous findings [103], this study proposes that a collection of multiple pollen species is the mechanism by which bees overcome elemental shortages in a particular species, which may contribute to explaining their foraging behavior [104]. The severity of habitat destruction and fragmentation as a bee stressor is maximized when one considers specialist solitary bees that unlike generalist pollinators only forage a single species or group of closely related species of flowering plants [105]. The impacts of habitat destruction on bee biology and ecology are divided into factors that affect the availability of floral resources for adult bees to forage [106], as well as factors that affect the ability of adult female bees to properly excavate and provision their nests [107] and the positive and negative impacts of urbanization on natural resources that are essential to bees [108].

Honey bees can be impacted by landscape composition, and recent studies have demonstrated that uncultivated forage was the most consistently related to colony survival and, in several cases, honey output [93]. Investigating temporal contributions of rapeseed and sunflower to honey bees, it was determined that flowering weeds provide up to 40% of honey bee diet composition during the two-month period between the blossom of these two crops [109]. As a result of this period simultaneously occurring with honey bee population peak in late spring, the vitality of available weeds and native plants as food resources during this timeframe is highlighted. Increased access to floral resource placement resulting from the implementation of environmentally favorable practices to promote bee forage in areas containing semi-natural habitats results in improved honey bee physiology, as by the measure of increased bodily fat mass and vitellogenin levels, as demonstrated in a recent study [94]. The examination of synergisms between the pollen species composition of honey bee diets and inoculation with Israeli acute paralysis virus (IAPV) revealed significantly lower mortality in bees fed poly-floral pollen compared to those only provided *Cistus* species pollen; bees fed *Castanea* species pollen exhibited intermediate mortality [52]. Elemental micronutrient concentrations were marginally higher in poly-floral and *Castanea* species fed bees compared to those fed either *Cistus* or no pollen; however, these values were not significant.

A co-inertia analysis of two different agricultural systems revealed increased wild bee species richness, abundance, and diversity in crop production sites rich in flowering grasslands providing bees with food resources, compared to livestock husbandry sites dominated by forage crops [110]. Bumble bees were also found to be less sensitized to intensive agriculture than solitary bees by measure of increased bumble bee and decreased solitary bee abundance in locations with high pesticide and nitrogen fertilizer input. Foraging in a landscape with fragmented patches, the solitary bees *Andrena flavipes* and *A. haemorrhoa* were determined to have larger body sizes by measure of inter-tegular distance in areas of decreased semi-natural habitat [111]. Studying the foraging traits of Meliponini species in a heavily deforested tropical landscape in southern Costa Rica revealed species shifts as a result of their foraging strategies [112]. Solitary-foraging Meliponini species were found to pollinate the greatest diversity of plant species that are most abundant when forest diversity is highest, and as a result, the populations of these Meliponini species suffer the greatest losses when plant diversity is decreased due to deforestation. Conversely, group-foraging Meliponini species were found to pollinate only a few plant species, and are the most successful in deforested habitats. Therefore, the deforestation in this landscape has shifted the survival success of both groups of Meliponini bees, favoring group-foraging species, and explaining the decreases in solitary species. Summarizing these studies, bee species richness is linked with increasing floral diversity in the surrounding environment. Questions concerning the quality and chemistry of floral food resources lead to the same outcome. A worldwide meta-analysis of previous studies on crop, weed, and wild plant nectar composition revealed significant differences in nectar quality between wild plants and both crops and weeds; while the region was found to have a slight but significant effect on nectar quality [113]. Chemical analyses of pollen and nectar from wild versus cultivated highbush blueberry (*Vaccinium corymbosum*) revealed a significant alteration characterized by domestication resulting in the reduced pollen chemical diversity of amino acids and caffeic acid esters [114]. Applying this data, *B. impatiens* consumption of the commercially available caffeic acid ester 3-*O*-caffeoylquinic acid reduced *Crithidia* species gut parasite infection at concentrations only found in wild, but not cultivated blueberry. While this indicates the negative impacts of crop domestication on floral nutrient quality and resulting bee health, large levels of genetic variation and heritability were documented for most of the floral traits. Therefore, the potential for selective breeding and subsequent incorporation of multiple blueberry cultivars for diversifying floral traits within a landscape is possible.

Landscape composition drives shifts in bee traits, for example, as demonstrated in a recent study [106] where bee community-level ecological traits shifted in response to the quantity of available habitat in southwestern France and southeastern Australia. In France, small, solitary, ground-nesting, polylactic, and late foraging species were positively correlated with larger amounts of and increased proximity to grassland, hedgerows, and woodland edges, while large, social, above-ground nesting, oligolectic, and early foraging species were negatively correlated with these landscape gradients. In Australia, small and ground-nesting species were positively correlated with canola (*Brassica* species) and agricultural crop landscapes, while large and above-ground nesting species were positively correlated with alfalfa and semi-natural habitats. The measurement of the effects of several habitat disruption variables on the response of different bee species has demonstrated that above-ground nesting bees were more negatively affected by agriculture practices leading to habitat fragmentation and fire, while below-ground nesting bees were more impacted by soil tilling practices in agricultural environments [107]. However, fire did not significantly impact below-ground nesting bees; above-ground nesting species were 15% less abundant in locations following recent burns and 8% more abundant in locations that experienced older burns, compared to unburned areas contained in the 19 worldwide datasets tested in this study. A positive relationship between above-ground nesting bees and increased regional forest cover, and less regional forest coverage and increased abundance of below-ground nesting bees has also been described [115]. Several studies have assessed the effects of a landscape on a particular bee species, generating mixed results. For example, *B. terrestris* in the presence of courgette (*Cucurbita pepo*) agro-ecosystems had high fidelity for nectar, but not for pollen [116]. Nonetheless, simulations of empirical data for nectar and pollen in the Bumble-BEEHAVE model revealed that early season courgette nectar increased the quantity of several colony endpoints. Therefore, this study suggests that while courgette can potentially boost bumble bee populations, other flowering plant species that are rich in pollen nutrients must be incorporated into the surrounding landscape to optimize bumble bee health. Placing *B. terrestris* colonies in maize fields reduced the diversity of pollen collected, which negatively impacted colony growth; conversely, placing *B. terrestris* colonies in oilseed rape fields had no impact on colony growth, by the measure of colony weight gain [117]. However, for oilseed rape, increased colony growth was only observed when high flower cover persisted at the end of the bloom timeframe. This finding highlights the importance of crop species and flower timing in providing *B. terrestris* with adequate food resources in intensively managed agricultural systems. At a specific nutrient level, it was found that carbohydrate regulation was the most important variable in maximizing *O. bicornis* body size and survival, where the larval pupation occurred only after ingesting a fixed quantity of carbohydrates, but variable amounts of protein [118]. The authors of this study propose that the wide range in larval protein consumption is a result of their dependence on maternal nest provisioning for obtaining protein, and hence explains *O. bicornis* vulnerability to changes in landscape floral composition.

Several studies have demonstrated the ability of gardens and other fragmented habitats in urban settings to support high densities of bees [119,120,121,122]. For example, it has been shown that while densities of bee nests were high in urban gardens and countryside hedgerows, fence lines, and woodland edges, they were low in countryside woodland and grasslands [120]. Bee sampling at 24 locations within an urban gradient in France over two years indicated that urban locations support a wide diversity of bee species, with the highest level of diversity (including the most parasitic species) occurring in intermediately urban locations [121]. Variations in the individual body size of *Anthophora plumipes* along a rural–urban gradient revealed more asymmetry in forewings for bees in rural environments than in urban environments [122]. Therefore, urban landscapes may provide *A. plumipes* with higher quality habitats as a result of wing asymmetry being associated with environmental disturbance and insufficient food resource availability. Assessing the effect of urbanization on the response of the bee guild-visiting creosote bush (*Larrea tridentata*) in fragment and continuous desert locations within the Tucson Basin of Arizona, it was concluded that differences in bee diet breadth and nesting biology best predicted bee species response to the size of a respective habitat site [119]. Declines in ground-nesting *L. tridentata* specialists were strong and significant in fragmented habitats smaller than 0.75 hectares, while those for ground-nesting generalists were weak and insignificant in these smaller fragments. In tiny fragments, these specialist ground nesters were typically absent, while the cavity-nesting *L. tridentata* specialist *Hoplitis biscutellae* was overabundant. *H. biscutellae* demonstrated the usual pattern of increased abundance of cavity nesters in the fragmented habitats observed in this study. The observation of wild bee species pollination of potted cucumber (*Cucumis sativus*), eggplant (*Solanum melongena*), and purple coneflower (*Echinacea purpurea*) plants in urban gardens revealed the dependence on a diverse bee community, thereby supporting the need for bee conservation in urban settings [123]. Non-crop flowering plants should be incorporated into urban gardens in efforts to support bee conservation. Analysis of the pollination of *Claytonia virginica* and *Polemonium reptans* growing in urban wooded lots revealed that while pollinators are essential for fertilization, local-scale habitat characteristics instead of habitat loss were the most predictive of pollinator abundance and diversity [124]. Specifically, increased amounts of bright light in a woodland area positively impacted pollinator visitation of *C. virginica* flowers, while a larger patch size of woodland area positively impacted pollinator visitation of *P. reptans*.

Conversely, a significant negative association between increased urbanization and bee and hoverfly (*Syrphidae* species) abundance and diversity was found in a study collecting voucher specimens at locations of similar habitat [108]. Exploring the effect of urbanization on managed versus feral honey bees has shown that pathogen susceptibility increases when honey bees are managed and placed in an urban setting. [125]. Feral bees displayed twice the number of immune genes as managed bees following an immunity challenge, while urbanization was postulated to support the transmission of disease agents. Surveying bee communities in an urban-to-rural gradient revealed that the observed sex ratio becomes more male-biased as urbanization increases [126]. This trend was primarily driven by declines in females of medium and large-bodied ground-nesting species. A study of the population structure of *Colletes inaequalis* in urban areas revealed a positive relationship between geographic distance and the genetic differentiation of individual nest aggregations [127]. This result suggests that higher levels of inbreeding are likely associated with increased urbanization, and highlights the vitality of the distribution of *C. inaequalis* nests within a landscape for abundance maximization.

It is currently known that habitat destruction is detrimental to bees by largely curtailing their access to quality and abundant floral food resources. New studies have further elucidated the vitality of plant species diversity by demonstrating differences in nutrient composition in the pollen and nectar stores of individual species. Taking these results to the next level, some studies have mapped the elemental components in the pollen of different plant species, and using this data, have related this output to the elemental nutritional needs of specific bee species. Other studies have similarly quantified the chemical composition of nutrients in pollens, and examined the factors driving these differences. We have also explored reports of certain agricultural crops greatly enhancing endpoints of colony fitness, while others do not. Other studies have described the varied effects of land fragmentation on bees, where cavity-nesting species are generally less impacted (and sometimes even increase in density) than ground-nesting species. Supplementing urban areas with flower mixes has been shown to help pollinators in some cases, and only in certain components of urban landscapes. Future work should combine the results of pollen and nectar chemical and elemental nutrient compositions with findings of optimal flower timing to maximize opportunities for bee health. Bringing these results to field settings and particularly to those that need restructuring from prior fragmentation will be a valuable contribution to ensuring that bees have access to the correct floral resources at the correct times.

## 3. Monoculture Plantings

Bees require a variety of nutrients in their diets comprised of carbohydrates (in the form of sugar from nectar or honey), amino acids (in the form of protein from pollen), lipids, vitamins, minerals, and water in the correct ratios for proper growth and development [35,128]. Agro-ecosystems have become increasingly intensified with large monocultures, which are defined as short massive gluts of the same species of flower dominating an agricultural landscape [17]. For example, the quantity of maize grown in the Central United States doubled between 2003–2010 [129], while California almond (*Prunus dulcis*) acreage increased from 323,749 hectares in 2012 [130] to 501,810 hectares by 2016 [131]. Monoculture exposure is proposed to negatively impact bees by depriving their access to a wide diversity of floral resources that are necessary to meet their nutritional requirements [132,133,134]. For example, in sampling stored pollen in honey bee hives, a study found that floral diversity was significantly linked to protein and lipid content, thereby suggesting that a consequence of monoculture foraging is insufficient protein and lipid dietary intake [101]. Limitations on honey bee growth as a result of diets lacking certain essential elements have been identified [102]. Since the pollen and nectar of many plant species are not rich in the complete spectrum of these elements, large-scale monoculture exposure may prevent honey bees from obtaining all of these elements. Therefore, it is advisable to incorporate a wide variety of flowering plant species into a cropping system that are placed within the realistic foraging distance of honey bee hives. However, this task is complicated, as different species of bees are attracted to different species of flowers, as demonstrated in a most recent study [97] where phacelia pulled social and managed species, compared to sunflower and wildflower mixes that pulled larger-bodied solitary species [97]. As a result, one must specify which pollinators are of interest and allocate floral species utilized by an entire guild of pollinators when designing pollinator strips in large monocultures. Moreover, not all monocultures are the same, as some can be worse for bees than others. In bumble bees, it was found that maize monocultures provided *B. terrestris* with low-quality pollen that negatively impacted colony growth, while oilseed pollen fostered colony growth, but only when mass flowering occurred at the end of the bloom period [117]. Other monocultures can provide bees with only part of their necessary nutrition requirements [116], where courgette fields provided *B. terrestris* sufficient nectar, but not pollen. Furthermore, differences in the chemistry of floral food sources and their resulting effects on pollinating animals have been documented across plant cultivars, classifications, pollens, and nectars. For example, in analyzing flower, nectar, and pollen methanol extracts for 31 plant species, it was determined that pollen contained higher levels of secondary chemicals (flavonoids, alkaloids, terpenoids, and phenolics) and 63% more chemical richness than nectar [135]. Sub-optimal larval growth in solitary bees such as that in *O. bicornis* resulting from insufficient intake of elemental nutrients [36,104] may be explained by the deprived consumption of secondary metabolites and other chemical compounds resulting from an inadequate diversity of pollen in parental food provisions. The domestication of highbush blueberry was correlated to a decreased chemical diversity of pollen and nectar, while *B. impatiens* consumption of a compound only found in wild cultivars reduced susceptibility to a *Crithidia* species gut parasite [114]. These results further support the notion that exposure to monoculture landscapes hampers bee health and vigor due to nutritional deprivation.

The importance of properly diversified dietary intake on honey bee adult and brood health, longevity, and survival has been demonstrated [128,136]. For example, it has been found that honey bees fed protein-free sugar syrup diets have lower protein levels in heads, smaller hypopharyngeal glands, and higher deformed wing virus (DWV) titers compared to honey bees fed with pollen or a protein supplement (MegaBee^®^) [136]. The assessment of transcriptomes of honey bees fed diets of pollen or sugar indicated that pollen-fed honey bees had activated nutrient-sensing and metabolic pathways and gene expression positively related to longevity and antimicrobial peptide formation [137]. Honey bees consuming sufficient quantities of quality pollen have increased resistance to pesticides as demonstrated in an analysis of the impact of pollen intake on chlorpyrifos tolerance [138]. However, other studies have indicated a widespread pesticide contamination of pollen, creating a dilemma given the vital role of pollen as the main source from which bees obtain protein. In a survey of honey bee colonies from apiaries located in six states within the United States, 79 pesticides and metabolites were recorded in pollen, and 56 were recorded in wax samples [139]. In all four years of the study, pesticides with 10 modes of action were detected, while nine additional modes of action were detected in one year. Therefore, this study concluded that synergistic toxicities of pesticides to bees arising from exposure to multiple modes of action in pollen and wax have a high probability of occurring. The residues of 18 different pesticides were detected in 62% of honey bee corbicular pollen samples collected from 53 Italian apiaries over a three-year period [140]. Feeding honey bee workers pollen contaminated with the fungicides pyraclostrobin and boscalid in cage studies and colony-level field studies resulted in bees consuming less fungicide-tainted pollen, digesting less protein, displaying lower ATP levels, and higher virus titers [141].

Native bumble bees blend pollen collected from native and exotic plants to meet their unique protein and essential amino acid nutritional needs [142]. This finding indicates that bumble bee flower choice is not random, and is instead driven by their specific nutritional needs. Bumble bee dependence on quality floral resources providing pollen with significantly higher protein content and quantities of essential amino acids is classically displayed in a comparison of the spectra of pollen collection by *B. terrestris*, *B. pascuorum,* and *A. mellifera* colonies in a field setting experimental design [132]. Limiting food resources available to *B. terrestris* colonies demonstrated that colonies are most sensitive to inadequate access to food resources during their initial development in the early spring [143]. Placing *B. impatiens* in three different ecological habitats facilitated the isolation of a strongly positive relationship between colony nutritional intake (calculated as the quantity of proteins, lipids, and sugars collected by the whole colony) and the number of offspring the colony produced (calculated as colony reproductive output) and overall colony size [95]. This finding demonstrates that while habitat does not impact the nutritional quality of collected pollen, proper colony growth and development are linked to consistent nutritional intake, and are thus a measure of the availability of appropriate nutritional floral resources within a landscape. Similarly, it has been suggested that *B. impatiens* foraging decisions are influenced by specific macronutrient ratios by means of a demonstrated preference for flowers with high protein to lipid ratios [144]. These results suggest that an abundance of floral resources with appropriate nutrients is imperative to bumble bee colony health, survival, and success.

Solitary bees also have similar nutritional requirements, for which most studies suggest that a departure from monoculture plantings is the best remedy [118,145,146,147]. For example, a range of seven to 1100 flowers or 0.9 to 4.5 flower heads is required to rear a single larva of 41 European bee species, depending on both species and host plant [148]. Dissecting the gut regions of *Nomia melanderi* revealed that 85% of bees contained pollen in multiple gut regions, indicating active feeding, and increased pollen feeding throughout the day [149]. *Ceratina calcarata* larvae provided with additional pollen consumed significantly more pollen, had larger adult head widths, and contained greater stored fats compared to those provided with reduced pollen [150]. Non-diapausing *M. rotundata* that eclose as adult bees in the same year as their larval development are problematic for the breeding industry of this bee, because non-diapausing females provision nests when floral resources are scarce [151], have lower reproductive success, disperse from alfalfa fields [152], and must chew through and kill diapausing nestmates while emerging from their nest [9]. Addressing this issue, a study investigated the impact of provisioning *M. rotundata* larvae with one of six diets differing in quantity of food (by weight) and quality (absence or addition of honey bee royal jelly) on entering diapause [152]. They found that larvae provided diets of decreased food quantity were less likely to enter diapause and weighed less as adults, while those provided diets of increased food quantity entered diapause more often and weighed more as adults. Interestingly, higher quality diets resulted in increased diapause, but significantly reduced adult weight. The incorporation of a competitive nesting bioassay comparing adult females from each of the six larval diets tested demonstrated the significant impact of weight where heavier females controlled nest sites for the most days. Future research should build on this preliminary study to develop a thorough and robust understanding of the stressors impacting *M. rotundata* given the vitality of this bee for alfalfa production [9,153]. Comparing *Osmia bicornis* and *Osmia cornuta* larval development on different species of pollen, it was concluded that *O. bicornis* developed well when consuming *Ranunculus* pollen but not *Echium* pollen, while the opposite was true for *O. cornuta* [146]. Larvae of both species responded favorably to *Sinapis* pollen, but not to *Tanacetum* pollen. These results pinpoint the physiological dependence of solitary bees on specific plant species for proper larval growth and maturation to adult bees. Using *O. bicornis* larvae as a model for solitary bees, a separate study found that compared to males, females require higher quantities of P, Cu, and Zn in their diets, and female fitness may be particularly linked to high P consumption [147]. Therefore, male and female eggs could be provisioned with different pollen mixes. As a result, it is integral that fertilized females have access to key plant species providing pollen rich in these elements to ensure proper male and female offspring development.

The importance of access to diverse floral resources for optimal bee nutrition, and the deleterious impacts of monoculture exposure on enabling bees to fulfill this requirement are well documented in the literature. New findings have shed light on the chemical and stoichiometric complexity of bee dietary needs, demonstrating that most pollen and nectar stores lack this complete spectrum. Therefore, it is postulated that many bee species compensate for this by mixing pollen from multiple plant species, thereby supporting the vitality of floral diversity from a metabolic perspective. Moreover, several studies have found that nutrient quality differs among cultivars, while specific floral enhancements are only suited for certain bee species. Additionally, monoculture crops differ in the quality of their nutrient provisions. Using this compilation of data, future work should build upon current practices of simply planting wildflower mixes alongside monocultures. To this regard, an investigation of realistic methods for designing monocultures that incorporate pollinator provisions adequately suiting the unique needs of all bees within an ecological guild (not just a selecting a group of common managed species) is warranted. A specific goal should be creating landscapes within crop layouts that incorporate the specific flowering plants of multiple managed and feral bee species. Breeding programs can create lines of crops and even wildflowers that are selected for traits influencing the quality of their pollen and nectar. Monocultures and surrounding areas equipped with floral resources rich in the diversity of elemental nutrients required for optimal bee health will help mitigate bee declines due to the removal of native plant species, while contributing to ecological and environmental restoration efforts.

## 4. Parasites, Pathogens, and Pests

Bees are naturally afflicted by a variety of parasites, pathogens, and pests. The vast majority of research has focused on honey bees and to a lesser extent on bumble bees; therefore, little information is currently known regarding solitary bees. Naturally occurring disease agents are known to control populations of their bee hosts within their native range, although most are not well understood. Several bee pathogens affecting bees (e.g., *Nosema ceranae*) have broader host ranges, while others (e.g., *Crithidia bombi*) have been found to be more specific [154,155]. The impacts of parasites, pathogens, and pests on bee health are briefly discussed below.

### 4.1. Parasitic Mites

*Varroa* mites inflict harm to individual honey bees and entire colonies as ectoparasites and by vectoring viruses such as DWV [156]. It is currently believed that *Varroa* mites are incapable of parasitizing non-*Apis* bees [17]. However, acute bee paralysis virus (ABPV) (believed to be vectored by *Varroa* mites [157,158]) occurrence in honey bees was found to significantly predict bumble bee infection, where feral bumble bees contained substantially higher infection levels than honey bees [159]. In another study, supplementing 246 DWV sequences confirmed that although DWV is an endemic honey bee pathogen, it has recently reemerged by the spread of *Varroa* mites [39]. The assessment of *Varroa* mite spread throughout the Hawaiian Islands showed a significant link to the prevalence, viral load, and strain of DWV [160]. Higher virus levels resulted in decreased strain diversity and a million-fold difference in viral load between *Varroa*-free and *Varroa*-infested areas [160]. Supporting these findings, a separate study identified a close statistical relationship between autumn colony infection with a specific DWV genotype (DWV-B) and overwinter colony mortality that did not hold true for either infection with diseases or springtime colony infection [161]. Similarly, inoculating individual bees with different DWV strains confirmed that DWV-B is the most virulent, and using post hoc qRT-PCR demonstrated the highest titer levels in the DWV-B treatment compared to the other strains tested at nine days post-inoculation [162]. Investigating impacts of DWV on honey bee behavior resulted in (1) significantly fewer DWV-inoculated bees surviving to forage age and (2) an average premature onset of foraging of 2.31 days, and an average life-expectancy reduction of 4.74 days [163]. Recently, a mutualistic symbiosis between *Varroa* and DWV, affecting cellular immune responses with NF-kB signaling, that enhances mite reproduction has been uncovered [40]. In a recent survey of the Varroa destructor virus-1 (VDV1) in 603 United States apiaries, 66% of honey bee pupae samples were infected, compared to a 2.7% infection rate from a 2010 analysis of 75 colonies [164]. Taking these findings into the context of non-anthropogenic interactions between feral and managed honey bees, it is proposed that these interactions explain the observed resilience of bee populations to *Varroa* mites in Africa and the Americas [165]. The elimination of human interference enables natural adaptation and resistance development in wild bee populations, which will in time filter into managed bee populations. This same scenario should be explored in bumble bees, given the rise in the utilization of a few domesticated species for pollination services on a global level.

### 4.2. Bacterial Disease

American foulbrood (AFB) (*Paenibacillus larvae)* is considered the most devastating disease of honey bee brood globally, and can spread rapidly, leading to colony failure if left untreated [154,166]. While current control tactics include the application of antibiotics (such as oxytetracycline and tylosin) and burning infected hives [167,168], the beekeeping community is largely interested in alternative control methods, given resistance development and efforts to decrease chemical application inside beehives. New management approaches include the usage of bacteriophages [43] where hive treatment with a cocktail of three bacteriophages resulted in complete protection from AFB. Discoveries of honey bee natural defenses inhibiting different strains of *P. larvae* growth such as the 10-hydroxy-2-decenoic acid (HDA) major fatty acid in royal jelly [169] and honey bee lactic acid bacteria [170] are promising and warrant further research.

### 4.3. Fungal Pathogens

*Nosema* species are obligate intracellular parasitic spores affecting both individual honey bees and entire colonies of *A. ceranae* and *A. mellifera* following consumption of their spores [171,172]. A high infection rate of *N. ceranae* in honey bee queens artificially inseminated with either the spores or semen of infected males has been observed, thereby indicating the potential for sexual transmission [42]. However, interestingly none of the eggs laid by infected queens were infected, thereby negating the subsequent vertical transmission in this exposure scenario [42]. Adverse effects on honey bee survival following inoculation with *N. apis* and *N. ceranae* have been demonstrated in the past [173]. *N. apis* inoculation resulted in significantly higher honey bee mortality compared to *N. ceranae* [174], which supports data suggesting *N. ceranae* dominance (resulting in colonies living with sub-clinical infections) over *N. apis* (resulting in colony death) [173].

Several *Nosema* species such as *N. bombi* and *N. ceranae* can also parasitize bumble bees [175,176,177]. Observing *N. bombi* spread in a managed colony of *B. auricomus*, a study isolated the highest pathogen loads in males and lowest pathogen loads in queens and older workers, and concluded that if the described increased male susceptibility to *N. bombi* substantially reduces the quantity of males available to fertilize queens, this may impact bumble bee colonies [178]. Further confirmation of the global spread of *Nosema* in bumble bees has been documented in other studies [46,176]. Since the importation of commercially bred bumble bees to South America has been linked with pathogen spillover to native species and their recorded population declines [49], these findings of *Nosema* spread may be explained by this scenario. Similarly, *N. ceranae* and *N. bombi* in *B. motivagus*, *B. haemorrhoidalis*, and *B. breviceps* have been found in bumble bee screening in northern Thailand [177]. These initial confirmations are of importance in light of recent literature revealing that bumble bee parasites are on the rise. *Nosema* DNA extraction from the museum specimens of five North American and one European species showed significant increase in *Nosema* presence from the 1980s to the late-1990s, which corresponds with high levels of *N. bombi* outbreaks in North American managed bumble bee stocks [48]. The genetic comparison of global *Nosema* populations led to the conclusion that there is no evidence of an exotic origin for *N. bombi*, thereby indicating that *Nosema* strains currently afflicting North American bumble bees were present prior to the commercialization of a few bumble bee species. Interestingly, low genetic diversity was found for North American and European *N. bombi*, while high genetic diversity was found in Asia where bumble bee breeding is non-existent, thereby suggesting that breeding has resulted in selection for a resistant *N. bombi* strain [48]. Using rRNA analysis on collected specimens of native and imported bumble bee species from Chile and Argentina enabled the isolation of several *Nosema* microsporidian species in 2% of hosts, and confirmed that all infections were present in imported species [50].

### 4.4. Insect Pests

The small hive beetle (*Aethina tumida*) is a scavenger pest of honey bee colonies that inflicts damage as a result of larval feeding that can destroy entire combs [179,180] or collapse colony nests [181]. Given the rapid spread of this pest to apiaries globally, a control plan that focuses on completely engaging all stakeholders and emphasizes appropriately compensating beekeepers for hive losses in a timely manner has been devised [182]. Cultural practices including incorporating sentinel apiaries to facilitate early pest detection and the capture of escaped beetles, and the burning of all infested equipment has been recommended to manage this pest [182]. Finally, bans on migratory beekeeping are recommended to prevent pest spread to wild colonies, and when infestations are recorded in feral colonies, containment procedures should be applied. Nonetheless, there are some recent advances in the biology of this pest. These include the discoveries of significantly higher infestation levels in areas of forest cover and high precipitation compared to areas of savannah and low precipitation [183], and its association with the yeast *Kodamaea ohmeri* throughout its entire lifecycle [184]. Genomic sequencing has revealed increased levels of gene families involved in insecticide detoxification, where the quantity of detoxification homologs are substantially different from honey bees [185], and an improved multiplex polymerase chain reaction (PCR) bioassay targeting the cytochrome oxidase subunit I gene, which improves upon previous internal mismatches among nucleotides [186].

Some work has investigated tactics for controlling other honey bee pests. Once considered a nuisance pest, the worldwide distribution of greater wax moth (*Galleria mellonella*) has greatly increased, while its potential to vector viruses to honey bees is of major concern [187]. Economic damage results from larval feeding on and tunneling through brood, wax, honey, and comb. Recent advances in control tactics include those that are biologically mediated, such as a *Bacillus thuringiensis* (Bt) strain, a group of parasitic Hymenopterans, and the application of sterile insect techniques. Future work in wax moth control should test the feasibility of these options to maintain populations at tolerable levels given the increased importance of this pest to beekeeping operations combined with a desire to minimize chemical usage in apiaries. Similar to *Varroa* mites, *Tropilaelaps* mites have a similar lifecycle, and were originally identified as being pests of honey bee species in Asia [188]. Concern has risen, given reports that two species (*T. mercedesae* and *T. clareae*) can successfully reproduce in *A. mellifera* drone and worker brood cells [189]. Therefore, beekeepers and bee scientists should become familiar with this mite’s biology and be capable of identifying it in hives. Future work should assess the ability of this mite to spread between colonies and infest entire apiaries, as well as its potential to vector honey bee viruses.

### 4.5. Trypanosomatid Parasites

Trypanosomes afflict bees by foragers contacting contaminated flowers and bees contacting larvae and infected surfaces [190]. Once inside a bee host, trypanosomes establish in the hindgut [191]. Phylogenetic constructs confirming distinction between two *Crithidia mellificae* isolates and a previously undescribed taxon in *A. mellifera* (*Lotmaria passim* n. gen., n. sp.) revealed *C. mellificae* presence in bee populations with *L. passim* predominance in *A. mellifera* worldwide [192]. Building upon this work, a PCR procedure was developed to distinguish between *C. mellificae* and *L. passim* in honey bees [193]. A survey of honey bees in different parts of New Zealand revealed high levels of infection with two genotypes of *L. passim* that remained consistent yearlong with low genetic variation [194]. The low genetic diversity of *L. passim* identified close genetic matches to samples from Europe, South America, and the United States, which suggests introduction from one of these sources. The mapping of *L. passim* spread and strain prevalence as demonstrated in this study is necessary for predicting future distribution and resistance patterns to minimize honey bee declines and their impact on the apiculture industry.

Several studies have documented the rise and spread of *Crithidia* species and their potential contribution to bumble bee species declines in different world regions. For example, a comparison of bumble bees foraging farms using commercially bred *B. terrestris* to those foraging farms that were not initially found lower levels of *Crithidia* species infection on farms where commercial bumble bees were present [44]. While these authors attribute this initial finding to a dilution effect resulting from the high abundance of *B. terrestris* on commercial farms, sharp increases in *Crithidia* species infection were documented in *B. terrestris* by season end, which are also attributed to the high density of managed bumble bees promoting rapid virus spread. Although this study does not link *B. terrestris* spreading *Crithidia* species to wild bumble bee species, it raises the possibility of this scenario, and justifies the need for the direct investigation of its occurrence. Building upon this work, the molecular analysis of two subspecies of commercially produced *B. terrestris* by [49] identified five parasites (including honey bee parasites) in 13–53% of colonies, and three additional parasites in pollen provided to these colonies as food. Adult bumble bee consumption of feces or pollen from commercial colonies significantly reduced survival, whereas bees that consumed pollen instead of feces developed significantly more *N. bombi* and *Apicystis bombi* infections, and 45% of bees fed pollen and 11% of bees fed feces that died seven or more days after exposure contained *Nosema* in their guts. Also testing honey bees, this study found that survival was significantly reduced following intake of commercial bumble bee feces, and large quantities of honey bees became infected with *N. apis*, *N. ceranae*, and *A. bombi* following exposure to either bumble bee feces or pollen. Feeding honey bee larvae pollen from commercial bumble bee colonies significantly reduced their survival compared to controls or larvae fed commercial bumble bee pollen that was previously frozen or microwaved. Specifically, 55% of the dead honey bee larvae fed pollen were infected with *Ascosphaera apis*, compared to 23% of those fed frozen or microwaved pollen. The capability of *B. terrestris* larvae infected with *C. bombi* to transmit parasites to adults demonstrates that broods are a route of colony parasite transmission [190], and further exacerbates the potential for this pathogen to rapidly spread throughout a colony. Since queen fitness is adversely impacted by *C. bombi* infection [195,196], the increased prevalence of *C. bombi* as demonstrated in these studies may be one of the causes of observed bumble bee decline. However, some bumble bee innate immune responses to trypanosome infections have been discovered. *B. impatiens* gut microbiota protection against *C. bombi* characterized by high microbiota diversity and large gut bacterial populations resulting in lower infection levels has been shown [197]. Genome sequencing of *C. bombi* and *C. expoeki* revealed concerted evolution aimed at improving their ability to infect hosts [191].

### 4.6. Pathogens Infecting Solitary Bees

Solitary bee pathogens have been studied given interest in using solitary bees as pollinators. *O. cornifrons* DNA sequencing for pathogenic microorganisms revealed pathogenic and apathogenic fungi and a hypothesized apathogenic bacteria strain in asymptomatic adult bees that might be disease carriers [45]. Similarly, a variety of viruses—including several that were previously unclassified—have been discovered analyzing meta-transcriptomes of eight species of wild social and solitary bees [47]. The presence of a variety of honey bee viruses and parasites in the majority of solitary bee species sampled within the vicinity of an apiary suggests that beehives may result in pathogen spillover to solitary bees, and that once infected, solitary bees may become a reservoir for these honey bee viruses [198]. Similarly, molecular confirmation through phylogenetic analysis revealed black queen cell virus (BQCV) spreading and the commonality of strains between managed honey bees and wild *Andrena* species in commercial fruit orchards [199]. Additionally, trait-tip analysis revealed no BQCV bee species host segregation by a monophyletic clade score, indicating that neither species is significantly clustered together, while virus levels in bee samples were associated by geographical location. Conversely, elevated levels of sacbrood virus (SBV) and DWV were found in *M. rotundata* that were not linked to their placement alongside honey bee hives for canola seed production [200]. Further, although SBV and ABPV were detected in a survey of adults in three different regions, they were only detected in the pupal life stage in one region, and co-infection amongst adults was correlated more with decreased pupal vitality than pupal infection levels. Similarly, in testing four families representing eight genera of solitary bees for five common honey bee viruses, a study determined that while virus detection was high in solitary bees, they were present in levels substantially lower than in honey bees [201]. Moreover, inoculating adults of *M. rotundata* and *C. inaequalis* with a mixture of common viruses that are lethal to honey bees had no effect on their short-term survival.

Exciting new findings regarding the dynamics of how maladies afflict bees should be discussed when considering the future direction of research in this area. Viewing the impact of parasites and pathogens on honey bees from a cognitive and neurobiological perspective, it is proposed that they impact on behaviors such as foraging [202]. Pathogen activation of the immune system and the subsequent inhibition of signal transduction or bodily energy distribution might explain this scenario. Specific examples include the alteration of neurotransmitter signaling genes following *Varroa* infection [203], which is thought to be phenotypically displayed as impaired honey bee navigation [204,205]. Diseases vectored by *Varroa* are also thought to cognitively impair honey bees; DWV interference with olfactory learning might stem from its inhibition of brain components imperative for foraging [206], while homing behavior is derailed by IAPV [207]. In a meta-analysis of parasite and pathogen impacts on social bee cognition, the impairment of several progressive behaviors has been identified [208]. Colony infection with *N. ceranae* can significantly reduce the quantity of foragers, which is compensated by the onset of foraging in immature bees [209] that have less developed cognitive perception and hence foraging ability [210], thereby reducing the amount of food stores in the colony [211,212], leading to the onset of foraging in more immature bees. This scenario can snowball into ultimate colony death. Analyzing studies where infection with *C. bombi* led bumble bees to take more time in flower handling and reject a higher number of flowers [213], and where honey bee infection with DWV reduced flight distance and duration [214], it is asserted that these behavioral changes are the result of parasitic alteration of bee motor behavior and host nutrient depletion from parasitic feeding, which increases host energetic stress and thereby inhibits the ability for infected bees to forage properly [208]. One study assessed non-associative learning studies where *V. destructor* infestation reduced honey bee response to an odor stimulus after sugar stimulation [215], and concluded that interference with synaptic transmissions as a result of infestation explain these outcomes [208]. The results of a recent study demonstrating similarity in the response of *N. ceranae* infected and non-infected honey bees and bumble bees to a sugar stimuli alone confirms that parasite infestation impairs the ability of bees to associate an odor with a sugar reward, which might similarly be explained by the impairment of neuronal signal transduction that is necessary to perform this task [216]. It is suggested that feedback loops connect honey bee disease with poor nutrition and vice versa, thereby viewing the drivers of honey bee maladies from a new perspective [217]. Providing bees with sufficient floral nutrients is proposed to amplify their ability to ward off certain pathogens. Conversely, certain pathogens have been associated with inhibiting bee nutrition; therefore, future research should explore these relationships in more detail.

Substantial work has explored the molecular underpinnings for a myriad of bee diseases, pathogens, and pests. Within this context, genomic understanding for viral and pest activity and replication can be used to devise future control tactics that will mitigate resistance development to current methodologies. Several studies have confirmed the ability for parasites to jump between bee species (including those belonging to different taxonomic families) and from managed species to feral species, which raises concern given the massive global trade of commercialized colonies for pollination events. Moreover, while the impact of maladies on solitary bees is less known, this area has received considerable attention (including viral examination at the genomic level), given the serious concern of their population declines. Finally, new discoveries have illuminated the ability for pathogens to alter bee cognition and neurological function, which might help explain their mode of action. Future investigation should build upon current initiatives to decode the molecular basis for pathogen function. To this regard, it would be useful to further devise pathogen and parasite-induced changes in gene expression that inhibit neurological signal pathways necessary for normal behaviors, health, reproduction, and survival.

## 5. Pesticides and Agricultural Chemicals

Increased pesticide application and the simultaneous introduction of new pesticide chemistries over the last decade has been proposed to contribute to recent large-scale bee declines [218,219]. Agricultural chemicals are designed to kill arthropod pests and are widely used in integrated pest management (IPM) programs. However, their acute and sub-lethal toxicity to *Apis* and non-*Apis* bees has been widely demonstrated [220,221,222,223,224]. Therefore, pesticides are considered a bee stressor, and modifications in their use that minimize bee exposure and impact are necessitated. Since most bee pesticide risk assessment research has focused on honey bees, while large data gaps exist for non-*Apis* bees, primary emphasis is placed on literature investigating the latter. Brief mention of efforts to determine the molecular basis for honey bee pesticide tolerance are discussed, and set a foundation for the direction in which future pesticide studies on non-*Apis* bees should go.

### 5.1. Apis Bees

Testing the toxicity of various insecticides to honey bees of different ages and genotypes revealed that Italian bees (*A. mellifera ligustica*) were overall the most sensitive, and honey bee sensitivity to phenothrin and naled was significantly correlated with bee age [225]. For phenothrin, sensitivity was significantly higher for three-day-old bees than for 14-day-old, 28-day-old, or 42-day-old bees, while for naled, the exact opposite effect was observed. These complete differences in bee sensitivity to individual insecticides highlight the importance of precisely testing each chemical of interest by demonstrating that the effects of exposure to one insecticide cannot serve as a surrogate for how bees will respond to another insecticide. Oral exposure to nonlethal doses of propiconazole (7 µg/bee) and a range of clothianidin doses (in a geometric series quantified by a factor of two ranging from 0.25 to 0.8 ng/bee) caused significant synergistic mortality in honey bees at 4 h and 24 h after treatment [223]. Simulating honey bee field exposure to binary pesticide mixtures, synergistic toxicity for mixtures of imidacloprid + tetraconazole, sulfoxaflor or oxamyl, and additive toxicity for mixtures of imidacloprid + acephate or lambda-cyhalothrin was discovered [226]. Significant decreases in forager survival were found following exposure to simulated tank mixes of iprodione, and synergistic effects resulted from iprodione + pyraclostrobin and azoxystrobin [227]. The quantification of neonicotinoid and fungicide concentrations in oilseed rape pollen showed large residue amounts in pollen samples collected by honey bees foraging these fields, as well as in pollen samples taken directly from oilseed rape flowers and adjacent wildflowers [228]. Conversely, exposing honey bees to clothianidin seed-treated canola posed low risk to health, development, and colony overwintering success [229]. Similarly, honey bee hive placement in clothianidin-treated oilseed rape had no negative effect on a number of colony endpoints, and was even positive for increasing brood and adult bee output [230]. Moreover, decreases in several diseases were recorded; collectively, this data suggest that a colony as a living organism is relatively immune to exposure with clothianidin seed-treated agriculture. Investigation of the molecular mechanisms honey bees use to detoxify pesticides has elucidated our understanding of differences in their resistance versus susceptibility to different chemistries, and even differences among chemistries within the same class. For example, one study found that the toxicity of three pyrethroid insecticides was greatly increased when bees were exposed to the cytochrome P450 enzyme inhibitor piperonyl butoxide, and somewhat increased by the carboxylesterase enzyme inhibitor S,S,S-tributylphosphorotrithioate. [61]. This study demonstrates that honey bees rely on cytochrome P450 enzymes to metabolically detoxify and tolerate pyrethroids. Exploring the metabolic basis for differences in honey bee sensitivity to N-nitroguanidine neonicotinoids (i.e., imidacloprid) versus N-cyanoamidine neonicotinoids (i.e., thiacloprid), divergence in cytochrome P450 metabolism was confirmed [62]. Specifically, by exploring the functional expression of the entire CYP3 clade of P450s, this study isolated the P450 gene CYP9Q3 that is highly efficient at metabolizing thiacloprid; however, it is negligibly effective at detoxifying imidacloprid.

### 5.2. Non-Apis Bees

Analyzing pesticide risk assessment schemes for *Bombus* species, several studies identify soil as being a high-risk substrate for both larval development and subsequent adult queen overwintering in underground nests [57,231]. Since *Bombus* queens have an extended lifespan and are vital to colony survival, thorough investigation of their exposure to pesticide residues and potential spread to their developing immatures within a colony are necessitated. Queens are expected to be highly vulnerable to pesticide exposure prior to and during nest construction as a result of foraging for their own food resources during this period [232,233]. However, since little is known about queen dietary and metabolic needs, it is difficult to assess their pesticide exposure from consuming contaminated pollen and nectar. Since bumble bee queens forage for food resources during this period, they are also more likely to come into direct bodily contact with pesticide residues emitted into the environment as dust particles from seed-treated crops or foliar sprays [57]. Further complicating this situation is our lack of data regarding queen nectar and pollen consumption post-nest foundation, when colony workers feed the mother queen. Moreover, accurately determining the movement of residues across soil and into an overwintering queen, pre-pupae, and colony nest is complicated by differences in soil composition and chemical-specific reaction and degradation properties. Since bumble bee adults are generally larger than honey bees and are covered in dense hairs, they will receive a lower dose of pesticide per unit mass at a given concentration compared to honey bee adults [57]. Different foraging behaviors characterized by bumble bees visiting two to three times more flowers and being active in cooler temperatures and harsher environmental conditions may increase their exposure to pesticides compared to honey bees. Although the larvae of both bumble bees and honey bees consume similar quantities of nectar per day, bumble bee larvae can consume upwards of 130 times more pollen, thereby potentially increasing their exposure to residues in contaminated pollen. Nectar and pollen fed to honey bees is aged and enzymatically altered, which may dilute residues; bumble bee larvae are fed raw unprocessed pollen and nectar; therefore, it might contain higher levels of pesticide residues. In conclusion, honey bees are not an appropriate surrogate for determining the risk assessments of pesticides to bumble bees.

Other studies based on this platform are as follows. Exposing *B. impatiens* colonies to flowers sprayed with field-realistic levels of chlorothalonil for a month resulted in decreased worker production, decreased total bee biomass, and lighter-weight queens [55]. *B. terrestris* chronic exposure to field-realistic doses of thiamethoxam increased worker foraging duration and reduced forager pollen retrieval [234]. Negative correlations were found between high orchard pesticide usage and *B. impatiens* colony growth measured by the production of new workers and males and worker thorax width [235]. The collection of five bumble bee species over three sampling periods in different landscapes revealed detectable levels of at least one of a diversity of agricultural chemicals in 61% of specimens, with boscalid being the most frequently detected, and farmland being the landscape containing the highest pesticide concentrations [236]. Studies such as [139] demonstrated high levels of pesticide residues in pollen and wax within apiaries, which may result in contaminated honey bees spreading residues to flowers and other environmental surfaces.

A meta-analysis of the literature on the sensitivity of honey bee versus other bee species to pesticides indicates that although there is high variability in the sensitivity of individual species, honey bees are generally the most sensitive [237]. However, findings showing solitary bees having drastically different pesticide sensitivities compared to honey bees [220], combined with remaining significant uncertainties regarding the routes of pesticide exposure to non-*Apis* bees [238], have given rise to increased research efforts to thoroughly assess the toxicity of pesticides to non-*Apis* and specifically solitary bee species that are commercially reared. A workshop of experts in different aspects of non-*Apis* bee biology has served as a springboard for devising protocols to specifically address these shortcomings [238]. Comparing the importance of different pesticide exposure routes to honey bees and solitary bees based on differences in their life history traits, several studies have concluded that exposure to pesticide dust and spray as air particles is of high concern for both groups of adult bees, while soil exposure is only of relevance for ground-nesting species such as *N. melanderi* and mason bees (*Osmia* species females) that gather mud to construct their nests. [60,231]. It is agreed that in most cases, adult honey bee pesticide exposure from contact with contaminated plant surfaces can serve as an accurate surrogate for solitary bees, with the exception of *M. rotundata* [231]. Since females of this species line their cells with leaf pieces that they have previously cut and carried with their mandibles, pesticide residues on leaves can result in adult oral and contact toxicity and larval contact toxicity as a result of developing inside a nest made of pesticide-containing leaves. Although larval pesticide exposure via nectar and pollen is of major importance for all bee species, differences in age, enzymatic alteration, and source of pollen and nectar consumed by honey bees versus solitary bees may impact the ability for their larvae to degrade and detoxify the pesticide residues that are found in these food resources [60]. A list of metrics related to foraging behavior and energy allocation during flight are currently used for the quantification of honey bee pesticide uptake by the oral consumption of pollen and nectar; however, since this information is largely lacking for solitary bees, it difficult to directly quantify pesticide consumption levels for these bee species. An assessment of topical pesticide exposure can be facilitated by the measurement of pesticide residues on honey bee bodies; since this information is largely unknown for solitary bees, future work should apply this metric to these species [239]. At a field level, scientists argue that understanding the degradation and physical properties of a pesticide will aid in predicting its likelihood to come into contact with bees and its capacity to impact specific bee species. This is of particular use for cavity-nesting solitary bees that have different interactions with an agro-ecosystem by consequence of their biology compared to social bee species. For example, only bees that use vegetative materials in nest construction will come into contact with systemic and translaminar pesticides, while products in soil may impact orchard bees that use mud to seal their nests [240]. Efforts have been made to transition to chemistries that are less harsh on the environment and or that only target specific insects, which has definitely benefited pollinators and other non-target arthropods. However, in farming systems such as alfalfa, where these products are used to control *Lygus* species, introduced pollinating *M. rotundata* may be exposed by default of their nest-provisioning biology where pieces of cut leaves are used to construct nests [240].

In examining stingless bees (Meliponini species), it is important to note that while chemicals in soil and the leaves and stems of seed-treated crops may pose low toxicity to honey bees, they may represent a major route of exposure for stingless bees given their usage of plant resins, mud, and wax in nest construction [59]. Brood production in Meliponines is similar to that of solitary bees, where an egg is oviposited on top of a mixed pollen–nectar food mass in a sealed cell, and unlike honey bees, because Meliponini larvae feed on large amounts of pollen. Also similar to solitary bees, stingless bee larvae consume raw and unprocessed pollen; therefore, they may be exposed to higher concentrations of residues than honey bee larvae fed aged pollen, where residues may have naturally degraded over time. These differences combined with the doubled development time for Meliponini larvae to reach adulthood expose Meliponini larvae to constant oral and contact uptake with toxicants in their food supply and nest-cell walls. Risk assessment schemes must account for these differences, and indicate that honey bees may not be an appropriate surrogate for stingless bees. Investigation of the literature on non-*Apis* bees that are important for crop pollination revealed five Meliponine species [241], which can potentially be used as surrogates for bee risk assessment studies. However, similar to solitary bees, data on stingless bee pollen and nectar consumption rates and subsequent pesticide uptake are relatively scarce, and even where they do exist, they only estimate minimum quantities. This dearth in data necessitates the major need for future research efforts.

Additional studies building on this premise are as follows. Feeding adult *O. bicornis* in flight cages sugar water laced with field-realistic amounts of thiamethoxam (2.87 µg/kg) and clothianidin (0.45 µg/kg) resulted in decreased reproduction characterized by increased mortality and male-biased sex ratios [242]. *O. bicornis* nest construction and provisioning was reduced adjacent to fields sowed with clothianidin + beta-cyfluthrin-coated seeds [56]. Clothianidin oral exposure was found to induce a delayed response in *O. bicornis* compared to *A. mellifera* and *B. terrestris*, which is based on *O. bicornis* having the lowest LD_10_ and LD_50_ values at 72-h post treatment [223]. Further, a clear synergistic effect by measure of 50% *O. bicornis* mortality at 96-h post treatment was found for exposure to clothianidin + propiconazole, compared with 6%, 3%, and 12.5% mortality at this timeframe for *O. bicornis* exposed to control, propiconazole, and clothianidin treatments respectively. Similarly, a negative correlation between *Lasioglossum* abundance and species richness and orchard pesticide application was identified [235]. However, this study interestingly showed a positive correlation between *Andrena* species richness and orchard pesticide application. This finding is most likely an indirect relationship whereby *Andrena* species flourish in an environment where there is less competition for resources as a result of *Lasioglossum* species decline due to pesticide exposure. It is also possible that *Andrena* species are more tolerant to the pesticides tested in this study than *Lasioglossum* species. In contrast, no effects have been found on development in dosing *O. bicornis* larval provisions with clothianidin (0 to 10 ppb) [243].

Recent investigation of the impacts of pesticide exposure to bees has largely emphasized molecularly-mediated functions of detoxification gene expression, the application of laboratory results to field settings, and a wider focus on solitary bee species. Stemming from findings resulting from an Environmental Protection Agency-sponsored workshop comprised of scientists from academia, private industry, and regulatory agencies discussing the exposure of non-*Apis* bees to pesticides (‘Pesticide Exposure Assessment Paradigm for Non-*Apis* Bees’), the notion that the responses of honey bees to pesticides cannot serve as the surrogate for how all other bees will respond to pesticides has become more widely accepted among the scientific community. As part of these outcomes, differences in pesticide toxicity being driven by differences in bee species biology and resulting environmental interactions have begun to become elucidated. Building upon current laboratory studies on non-*Apis* species, future research efforts should map their interactions with pesticide-contaminated substrates related to their ecology (for example, leaf pieces in an alfalfa field for *M. rotundata*, and soil for *Osmia* species). A new paradigm as proposed by a group of bee researchers view pesticide impacts on pollinators as a feedback loop, where pesticide use influences subsequent pesticide exposure and ultimately pesticide effects [244]. Assessing the entire spectrum of the inherent costs and benefits of pesticide use from this perspective may facilitate a more cohesive platform where the decision process to use a particular pesticide involves a thorough assessment of all the potential downstream effects. The future of scientific research concerning the toxicity and impacts of pesticides to bees should incorporate (a) complete bee–ecological interaction analysis, (b) efforts to illuminate the molecular basis for pesticide detoxification (particularly in non-*Apis* species), and (c) the use of nano-formulated pesticide applications [117] that precisely control and time pesticide release around pollinator environmental exposure.

## 6. Human-Mediated Bee Movements

Anthropogenic actions related to the domestication of a handful of bee species for commercial pollination are indicated as contributing to bee stress by increasing disease spread amongst domesticated bees and pathogen spillover from domesticated bees to wild bees [39,49,74].

Introductions of honey bee species to non-native ranges have resulted in negative outcomes. For example, the importation of *A. mellifera* colonies to Brazil and subsequent escape of virgin queens led to interbreeding with feral *A. mellifera* species, resulting in the hybridization of highly aggressive “Africanized” honey bee strains [245]. The importation of *A. mellifera* into the native range of *A. cerana* has resulted in intermating between the two species, rendering inviable offspring and possible queen thelytokous parthenogenesis [246]. Reviewing human-mediated actions related to the spread of honey bee pathogens [247], researchers have suggested that the massive transport of colonies to California for almond pollination is likely to increase pathogen and pest spread between colonies [74,75]. For example, DWV vectored by *Varroa* mites has spread worldwide following international honey bee trade [39], while migratory beekeeping in the United States and Australia has been proposed to favor the spread of pathogens such as chalkbrood (*A. apis*) [248]. The transportation of honey bees for crop pollination also increases the prevalence of a fungal pathogen (*Nosema ceranae*) in honey bees [249]. A field experiment comparing commercial and experimental honey bee hives that were either stationary or migratory found significant decreases in lifespan and increased oxidative stress for bees exposed to migratory treatments [250]. Ecological assessment of the impact of beekeeping on plant–pollinator networks in an isolated island system revealed structural modifications characterized by a loss in diversity of wild pollinator taxa interactions with plant species as a result of dominance by honey bees [251].

The commercialization and global distribution of managed bumble bees such as *B. terrestris*, which have a high capacity to naturalize and establish in non-native habitats [76], has raised concerns regarding the spread of non-native species and non-local genotypes into intact ecological communities [76,245]. For example, *B. terrestris* mating with native *B. hypocrita* and *B. ignitus* in Japan has resulted in non-viable offspring [252]. While multiple subspecies of *B. terrestris* have been classified, only one is commercially bred, leading to non-native subspecies introductions in western Europe [253]. Evidence of a non-native *B. terrestris* subspecies outcompeting native subspecies in foraging and reproduction has been demonstrated [254], thereby validating this concern. qPCR screening of managed adult *B. impatiens* detected a 45% infection rate of one or more pathogens, warranting more rigorous screening protocols of commercial colonies to prevent pathogen spillover to native bee species [255].

The transportation of bees across different regions has potentially contributed to transmitting pests and pathogens as well. For instance, the presence of *Ascosphaera* species fungal spores in *O. cornifrons* adult bees indicates the transport of these fungi within the bees themselves from their native habitat in Japan to their introduced regions in eastern North America [45]. The distribution of commercially bred Meliponini species bees throughout Brazil is of concern for endangered species [256] such as *M. capixaba*, which might be at risk for a loss of genetic identity by means of hybridization [257], and which has been documented to interbreed with domesticated *M. scutellaris* [258]. Although it is currently unknown whether the human-mediated movements of stingless bees in Australia are responsible for the genomic extinction of rarer species through interbreeding [259], species including *T. carbonaria* and *T. hockingsi* are capable of rendering viable hybrid offspring in native and introduced habitats [260,261]. The transportation of bees could also cause physiological changes and stress in bees. For instance, long-distance transportation affects the development of food glands in honey bees (*A. mellifera*) [262]. In a recent study, long-distance transportation was found to cause temperature-related stress and population loss in honey bees [263].

A number of studies have demonstrated cases of negative outcomes when bees from different regions mix as a result of human-mediated actions. Much of the recent literature has focused on widescale reports of pathogen spillover from imported domesticated bumble bees to wild *Bombus* species. However, some reports have demonstrated similar scenarios occurring in solitary bees. Given the increased concern of solitary bee declines, more work is warranted in this area. Additionally, studies demonstrate that the installation of apiaries can drastically increase the competition imposed on native bee species for food resources [251]. This outcome, combined with the negative impacts of honey bee foraging on the pollination and fruit set of native plant species described in this study, brings a new dimension of the effects stemming from human bee movements to light. Future research should ask similar questions and build upon this study by testing the impacts of commercialized honey bees and bumble bees on native bee–plant ecological networks at different global locations and at a larger scale.

## 7. Increased Competition for Limited Resources

Ascertaining the impact of competition between bee species for limited floral and nesting resources on resulting species richness and diversity is relatively difficult [17]. However, some evidence does indicate that the increased competition in ecological communities is enhanced by introductions of large numbers of non-native species [77].

Through quantifying floral resource consumption in rosemary (*Rosmarinus officinalis*) and thyme (*Thymus vulgaris*) plots combined with floral resource density and bee diversity, it is concluded that resource consumption was primarily driven by honey bee visitation and marginally by bumble bee (*B. terrestris*) visitation [79]. This study also showed lower wild bee diversity in plots closer to apiaries, and concluded that honey bees affect wild bee diversity and abundance. Similarly, by introducing honey bee hives to an isolated plant–pollinator network, a separate study quantified significantly fewer fruits and seeds on *Echium wildpretii* and *Spartocytisus supranubius*, which were commonly foraged by honey bees in this project [251]. Interestingly though, a significantly higher fruit-set was observed for these two-plant species, which may be a result of their receiving higher, although less effective honey bee visitation. Furthermore, *S. supranubius* plants in closer proximity to apiaries produced significantly fewer fruits and seeds, as well as heavier seeds compared to those growing farther away from apiaries. Conversely, an analysis of the interactions between Africanized honey bees and Meliponini species competing for honey–water resources demonstrated rare and mild *Apis* bee aggression toward Meliponini bees; however, *Apis* bees were found to display high aggression toward each other [264].

Placing red mason bees (*O. bicornis*) in treatment cages with honey bees containing flowering plants resulted in reduced *O. bicornis* flower visitation and reproduction in the presence of honey bees, with *O. bicornis* niche breadth diminishing as the honey bee quantity increased [78]. It was calculated that a strong beehive collects the same amount of pollen that is necessary to produce 100,000 progeny of an average-sized solitary bee (*M. rotundata*) [265]. Using this metric, this study predicts that a typical apiary containing 40 colonies collects the equivalent of four million solitary bee larval pollen provisions over an average summer, thereby directly competing with and negatively affecting native wild bees.

Although it is difficult to precisely determine the impacts of resource competition on bee species richness diversity and abundance, several studies suggest that introductions of managed bees can drive increased competition with native bee species. While the findings of these studies are controversial and warrant more investigation, they are essential, especially when focusing on rarer non-*Apis* species that are of conservation concern. It would be interesting to map out the long-term impacts of resource competition on the margins of where different ecosystems meet. A prominent example of this might be the field edge of an agro-ecosystem where an orchard intersects with surrounding woodlands.

## 8. Climate Change

Climate change as defined by global warming combined with erratic weather conditions has been proposed to affect the symbiotic interactions of bees and the flowering plants they pollinate; however, the specific impacts of this variable are not well understood [17]. Mismatches between the timing of adult bee emergence and the onset of flowering is one concern, although most ecological data suggest parallel shifts between bee and floral species [266,267]. Increased temperatures changing distributions of flowering plants and curtailing the natural ranges of bees by shifting them further north and to increased elevations is another concern [268,269].

Linear changes between bee and flowering plant phenology in the northeastern United States over a duration of 130 years were demonstrated [270], while long-term consistency between early bumble bee foraging and spring flowering plants in Russia exists [271]. Concurrent linear trends exist for increased temperatures and earlier flowering dates [272] and increased temperatures and earlier first appearances of honey bee [273] and bumble bee spring flight times [274]. Conversely, the earlier flowering of bumble bee queen-dependent *Corydalis ambigua* driven by earlier snowmelt has created mismatches between flowering and bee appearance, which is not impacted by earlier snowmelt [81]. The timing of floral resource availability affects the growth of *B. vosnesenskii* colonies; however, no impact on colony reproduction was seen [80].

Analyzing specimens of *B. balteatus* and *B. sylvicola* collected over a 40-year timespan, researchers have shown significant shortening of tongue length, which has been attributed to a reduced density of flower species containing deep corollas at lower elevations resulting from average increases in summer temperatures [275]. Assessing bumble bee species distributions over a 20-year duration, a study determined uphill shifts and lower limit retractions in species occurrence, combined with a simultaneous temperature increase [268]. Conversely, using various climatic areas, a separate research project concluded that areas containing less temperature warming (the southernmost locations), higher rainfall, lowest water deficit, and wider forest cover contain the highest bee diversity [276]. According to this study, microclimates containing these conditions will serve as the best reservoirs of bee diversity in the future.

Changes in climatic patterns driven by global warming are an additional area of concern given their potential to stress normal bee–ecological interactions. Although in most cases parallel shifts have been identified between earlier flower bloom and bee pupal eclosion, some instances of mismatches have been found [81,267,269,270]. Differences in floral composition mixes over several decades have revealed adaptations in bumble bees, which are characterized by shorter flower corolla lengths leading to shorter bumble bee proboscis lengths [275]. Also, the elevation and latitude movements of floral resources have been correlated with similar shifts of bumble bee species distribution following this pattern [268]. While climate change as a bee stressor is often overlooked compared to other heavily studied drivers of bee declines, future work should incorporate its role in changing landscapes and resource availability and its subsequent impact on bees. Emphasis should be placed on solitary species as opposed to massively reared domesticated species used for pollination.

## 9. Interactions between Multiple Stressors

Bees and other pollinators are commonly exposed to multiple stressors through ecological interactions with their environments. Therefore, it is plausible to hypothesize that exposure to multiple stressors results in additive or synergistic interactions exacerbating detrimental effects to afflicted bees [277,278,279]. We have isolated four separate interactions between multiple bee stressors: exposure to multiple pesticides, exposure to pesticides and diseases, exposure to pesticides in settings containing insufficient nutrients, and exposure to diseases in settings containing insufficient nutrients.

An assessment of the chronic oral toxicity of pesticides found in hive pollen and wax to honey bee larvae revealed synergistic toxicity for binary mixtures of chlorothalonil (34 mg/L) + fluvalinate (3 mg/L), and synergism between chlorothalonil (34 mg/L) + coumaphos (8 mg/L) [82]. Analysis of the impact of *B. terrestris* oral dietary exposure to imazalil in binary combinations with four insecticides demonstrated synergized mortality following the ingestion of imizalil + fipronil, cypermethrin, or thiamethoxam, but not imidacloprid; however, there was no synergistic impact on the *B. terrestris* feeding rate [280]. Testing the oral toxicity of a range of pesticides alone and in binary combinations to *A. mellifera*, *B. terrestris*, and *O. bicornis* following prolonged exposure (240 h) revealed additive toxicity following exposure to most mixtures, slightly increased toxicity following exposure to clothianidin + propiconazole, and a weak antagonistic interaction following *B. terrestris* and *O. bicornis* exposure to clothianidin + dimethoate [281].

Clothianidin contact negatively impacts honey bee immunity in promoting DWV replication by enhancing the transcription of a gene inhibiting NF-_k_B activation [41]. Significant correlation exists between neonicotinoid exposure and increased *Varroa* and associated viral pathogen levels [282]; indicating colony proximity to treated fields can subtly increase pathogen infestation. Exposure to sub-lethal concentrations of thiacloprid and microbial pathogens resulted in additive toxicity between thiacloprid and BQCV on larval survival, and synergistic toxicity between thiacloprid and *N. ceranae* on adult survival [283]. Chronic honey bee brood exposure to Sylgard 309^®^ and an inoculum containing four viruses showed synergistic toxicity characterized by mortality at larval–pupal molts [284].

Limiting honey bee exposure to nectar and or only providing bees nectar with low-sugar concentrations combined with exposure to sub-lethal doses of clothianidin (^1^/_5_ LD_50_) and thiamethoxam (^1^/_25_ LD_50_) synergistically reduced survival by 50% [84]. Conversely, in hive treatments with tau-fluvalinate, Fumagilin-B, and chlorothalonil had no significant effect on honey bee worker wet weight, protein, and carbohydrate quantities compared to control hives [285]. Evaluating the impact of consuming pollen containing the fungicides boscalid and pyraclostrobin on honey bee workers at high concentrations in cage studies and field-realistic concentrations in hive studies, it is pointed out that while hemolymph proteins did not differ between treatments, bee consumption of fungicide-laced pollen resulted in lower ATP concentrations and increased virus titer levels [141].

Honey bee colonies fed protein supplements had higher BQCV infection rates, *Nosema* quantities, and queen losses compared to colonies provided with natural forage, which contained higher soluble protein and amino acid levels [83]. Colonies in apiaries surrounded by diverse floral habitat exhibited decreased *V. destructor* and *N. ceranae* levels compared to those that were not [286]. Similarly, honey bees feeding on a diversity of pollens from multiple plant species have upregulated innate immunity and decreased mortality rates from *N. ceranae* infestation [287] and IAPV infection [52]. Pathogen infestation has also been found to inhibit or limit the ability of bees to obtain proper nutrients. For instance, where colonies infested with *Varroa* were shown to have decreased lipid stores [288], and where pupae infested with *Varroa* had significantly lower protein levels, increased free amino acid concentrations and decreased adult eclosion weight were also detected [289].

Traditionally, studies have investigated the impacts of a single stressor on bees (i.e., pesticide exposure, disease inoculation, or nutrient restriction). However, as described in the above studies, multiple stressors are often present in bee environments and can potentially inflict bees at the same time. Therefore, future work should strive to simulate realistic scenarios of how bee stressors can interact and exacerbate bee declines. Especially when these studies are conducted in field settings, they may elucidate feedback loops between stressors that depict how one stressor drives another. Research demonstrating the effect of honey bee pesticide exposure on viral gene replication [41], and demonstrating how poor nutrition increases honey bee susceptibility to viruses and vice versa [217] equip us with information that can be used to prevent these toxic interactions in advance. For example, genetic engineering may be used to modify the genes within a virus that respond to pesticides, or prior knowledge of a bee viral infection will call for a change in pesticide selection or application timing. Similarly, knowledge of bee-specific nutritional needs for viral immunity can be translated into floral species selection when designing pollinator strips or other wildflower plantings; conversely, preventative actions can be taken to control a nutrition-inhibiting virus at initial onset, including providing infected bees with additional food resources in cases where they are commercially managed. A recent report views pesticide impacts on bees from three interlinked dimensions driven by initial pesticide application and the complete environmental fate of a pesticide, including its impact on pollinators [244]. Future work on bee stressors should take a holistic approach where all the components of a bee stressor and its potential interactions with other bee stressors and environmental stimuli are thoroughly mapped out. Investigation in this direction will illuminate interconnected underpinnings of how stressors interact to afflict bees in ways that are currently not understood.

## 10. Initiatives to Support Bees

Concern over widescale bee declines has led to public and private initiatives geared toward conserving their populations. We divide these initiatives into two categories: wildflower plantings aimed at providing bees with sufficient floral resources, and modified pesticide application emphasizing alternative timing, less usage, or chemicals of biological origin.

The creation of bee-friendly habitats alongside train stations, on building roofs, and in public parks has enabled Amsterdam to increase its bee diversity by 45% since 2000 [88]. For example, the Zoku hotel has designed a rooftop garden and dining area containing a variety of flowering plants pollinated by specialist native bees, while a recent project called Honey Highway successfully planted wildflowers alongside a new road. Organizations such as the Bee and Butterfly Habitat Fund and Seeds for Bees plant pollinator-friendly seed mixes on agricultural lands comprised of native plants [85,290]. Governmental programs including the Dutch Bee Strategy, the English National Pollinator Strategy, and the International Pollinator Initiative promote pollinator health by bringing together beekeepers, growers, pesticide applicators, and other stakeholders [86]. However, simply initiating these programs is not enough. For pollinator enhancements and restoration projects to function long term, thorough engagement of community members such as citizen scientists and youth is essential, in conjunction with the goals of ecological restoration [291]. Additionally, many questions regarding the optimal design of conservation gardens for serving the specific needs of bees and other pollinators lack sufficient answers (especially within urban settings). Studies that demonstrate this point include those showing that pollinators are most influenced by components within a garden compared to the surrounding landscape [292], as well as the findings of another study indicating that many restoration initiatives solely focus on plant species restoration without specific consideration for pollinators [293]. Careful planning with precise goals for addressing the unique dietary, nesting, and other ecological needs of bees that directly involve community members is essential for pollinator gardens to be successful long term.

While initiatives to aid honey bees have widely caught public perception and media attention, protecting honey bees may not be the most important concern for bee conservation. Specifically, honey bees are not International Union for Conservation of Nature (IUCN) red-listed for being at risk of extinction. Moreover, honey bees are imported to North America in large quantities [294], and are commercially bred on large scales for pollination events. Meanwhile, although a small number of bee species native to North America are classified as being at risk for extinction [295], we still lack the required data to determine the population status of most native bee species [296]. Therefore, findings of pollinator restorations only serving a few species of common or managed bees, while largely ignoring native species, are concerning. For example, it was found that farms providing floral resources only attracted honey bees and bumble bees, while farms that did not attracted a diversity of bee and wasp species [297]. Considering these outcomes in light of the results of a recent study [298] where the presence of high-quality floral habitat placed within foraging distance significantly increased the survival of multiple colony lineages in three bumble bee species creates a paradox. Does the implementation of floral resources harm native bee species more than doing nothing, given their attraction to a handful of common species? Do pollinator gardens create situations of increased competition for food resources that leave native species worse off? Data generated from several other studies investigating the functionality of pollinator plantings agree with these questions. The examination of pollinator usage of sown plants in agricultural systems revealed that only 25 of 72 observed solitary bee species meaningfully utilized the flowers of these plants, while most solitary species instead utilized the flowers of native plant species that were not present in pollinator restorations [299]. Assessing the pollen usage of six cavity-nesting solitary bee species within farming landscapes, it was determined that only 15 of 32 plant species in implemented wildflower mixes were within the foraging proximity of these species, while *R. acris* was the only one of these species present in their pollen collections [300]. Analysis of pollen samples revealed that of 23 identified plant species, *Rosa canina* was most represented, demonstrating that this species can serve as both a nesting substrate for *M. rotundata* and a food resource for solitary cavity-nesting bees. Plant species mixes implemented into future pollinator gardens should specifically be geared toward serving the unique food and nesting needs of native bee species guilds instead of managed species. Since covering an entire bee ecological guild may be a lofty proposition, efforts should hone in on key solitary species such as the cavity-nesting species [300]. Future outreach efforts should work to shift the public perception of bees to this domain as well.

Findings of numerous studies on societal general perceptions of bees indicate the need for improved extension work that educates communities on the vitality of bees to ecosystems and food production. Specifically, efforts are needed to (a) promote bees as pollinators instead of stinging insects, and (b) distinguish bees from other insects. Questionnaire respondents ranging from primary school to university students with novice to beekeeper experience with bees indicated that while they were very interested in bee conservation, they associated bees with danger, given the risk of being stung [301]. An appropriate introduction of basic bee biology and removal of incorrect information regarding bees is believed to increase public perception of bees. These efforts are timely, given the current public interest in bee conservation. However, as described in a survey, honey bees occupy the majority of public perception on bees, given that only 14% of respondents correctly estimated the quantity of identified bee species in the United States, while many respondents could not discern bees from other insects when presented a series of photos [302]. Similarly, a survey of adolescents revealed limited knowledge about bees and common confusion with other insects [303]. Survey results also indicated that adolescents engaged in gardening and other outdoor activities tended to have improved knowledge of and attitudes regarding bees. Therefore, while extension and outreach have been paramount to promoting the importance of bees at a time of major bee declines, their message must be modified from honey bees to native bees. Educating youth should be a particular focus.

Extension has also delved into informing the public on basic steps that they can perform to promote bee health and conservation. Efforts to educate residents and businesses on alternative methods for controlling pests on private land that minimize the use of chemical pesticides are incorporated into Amsterdam’s goal of protecting bees and the natural environment [88]. Modifying the timing of pesticide application through for example spraying neonicotinoids 10 days before apple blossom, provides ample control of rosy apple aphids (*Dysaphis plantaginea*) in a method that drastically decreases pesticide residues in bee-foraged nectar and pollen [87]. A thorough analysis of insecticide options for organic farming operations by the Xerces Society for Invertebrate Conservation recommends the use of non-toxic Bt, *Cydia pomonella granulosis*, garlic, and kaolin clay, and moderately toxic boric acid, limonene, neem, and ryania/ryanodine to minimize harm to bees [304].

Considerable efforts have been made to aid bee conservation. These include the implementation of pollinator strips in agricultural settings, pollinator gardens, and beehives in urban settings, as well as efforts to educate the public on the vitality of bees. However, the vast majority of these initiatives have focused on key species such as honey bees, which are commercially bred, instead of native bees, some of which are at risk of extinction. Evaluations of pollinator plant fidelity reveal that native bee species most heavily rely on wild plant species, as opposed to those plant species that are typically incorporated into implemented pollinator floral mixes for their nectar and pollen food provisions. Therefore, there is need to restructure pollinator wildflower mixes to serve the needs of native bees instead of a handful of managed or common species. Surveys of public perception on bees show that while society is overall interested in promoting bee conservation, most people associate bees with honey bees, and therefore do not appreciate the diversity of bee species. Some extension efforts have been made to educate agricultural growers and urban citizens on IPM and other pest control tactics that will minimize their impact on bees. In summary, studies indicate that while considerable strides have been made over the last decade to aid bees, they have been skewed to focus on the wrong species. Therefore, future pollinator restoration projects and extension and outreach activities should shift their objective toward native bee species that are most in need of conservation initiatives.

## 11. Conclusions and Recommendations

A multitude of stressors impacting bee abundance and overall health have been identified over the last decade through studies investigating causative factors for their widescale declines. In highlighting recent advances in bee decline research, this review identifies eight key dynamics contributing to bee stress and methods for reducing their impact on managed and feral bees. However, specific considerations must be taken for managed bees versus wild bees, given that the stressors impacting each of these groups are different. Ideally, our hope is that efforts to aid bee populations will address all of these dynamics given that they are interlinked, as we have demonstrated in the section exploring interactions amongst multiple stressors affecting bees simultaneously. Additionally, removing one stressor while leaving others in place will not completely remedy bees. For example, if wildflower plantings are maximized but pesticide application is not modified, or vice versa, bees could potentially still be harmed. Similarly, if managed honey bee colonies are kept out of monocultures, while no effort is made to diversify floral resources, or vice versa, native bee species might be hurt.

Managed bees (honey bees and domesticated bumble bees) are primarily stressed by nutritional imbalances and the rapid spreading of diseases and associated pests. Although major efforts have been made to provide bees with wildflower plantings, several studies show that optimal nutrition is driven by meeting specific protein, lipid, carbohydrate, and even elemental requirements [102,144]. Therefore, floral mixes should be tailored to contain plant species providing nectar and pollen that are rich in these resources. Supplying managed bees with the proper nutrient resources will additionally aid in their innate immunity to viral infections [52]. *Varroa* mites are considered the most important problem for honey bees, especially when considering their symbioses with honey bee diseases, as in [40]. Other maladies afflicting honey bees including American foulbrood and small hive beetle have rapidly spread and are becoming of greater importance. Rapid upticks of parasites such as *Nosema* species (which traditionally were a parasite of *Apis* species) have been identified in *Bombus* species [48], which are believed to be a result of spillover from large-scale bumble bee domestication [44,49]. It is believed that these stressors are in part driven and exacerbated by the human-mediated actions of mass rearing of domesticated species and their global dispersal for pollination events. Exposure to pesticides has also been identified as a stressor of managed bees. While a myriad of laboratory and some field studies have tested the toxicity of pesticides on select bee species over the past decade, research efforts should shift to testing exposure to specific species of interest, as opposed to using honey bees as a surrogate. Evaluation methods should examine exposure routes based on species-specific environmental interactions [57] for domesticated bumble bees. A molecular-level analysis of pesticide detoxification mechanisms [61,62] should be more heavily prioritized. Future work should devise mechanisms for maintaining managed bees in more humane methods that minimize their impact on the surrounding environment, and consider the conservation needs of other pollinating species.

Wild bees (many of which are monolectic or oligolectic pollinators) are largely stressed by the loss of the plant species that they rely upon for food [112]. Fragmentation has also been found to negatively impact ground-nesting species, while promoting cavity-nesting species [115]. Apiaries in natural areas have also been labeled as a driver of wild bee declines, due to flooding ecosystems with honey bees that outcompete native bees for limited floral resources [251]. Rises in viruses inflicting wild bees have been correlated with pathogen spillover from interaction with managed bees, as in [198,199]. However, much data regarding the biology and extent of wild bee viruses is lacking. Therefore, future research should investigate this dynamic. There is growing agreement amongst the bee science community for separate pesticide toxicity bioassays that test specific non-*Apis* species of interest, given their vastly different biology and ecology from honey bees. Moreover, it is agreed that the susceptibility or resistance of honey bees to a particular chemistry should not serve as the surrogate for non-*Apis* species, while toxicity studies should examine realistic routes of exposure based on species-specific ecological interactions, as discussed in multiple studies [59,60]. Given the relative shortage of data on stressors driving wild bee declines (compared to managed bees), a shift of future research initiatives toward meeting this goal is vital for realistically understanding the impact of drivers on wild bee declines and ensuring their conservation.

Recent work has pinpointed combinations of bee stressors acting together, indicating that the ultimate effects of stressors on bees is the result of synergisms from their interactions. Here, we have identified four groups of combined bee stressors: exposure to (a) multiple pesticides [281], (b) pesticides + diseases [41], (c) pesticides + insufficient nutrition [84], and (d) diseases + insufficient nutrition [52]. These studies suggest that the combined effects of stressors may pose greater dangers for bees than their single effects. Contrary to traditional methods, these new findings view bee encounters with stressors as a measure of interactions among the multiple factors they are exposed to during normal ecological interactions with their environment, thereby creating a new paradigm for the study of factors driving bee declines. Therefore, future research should embrace opportunities to test realistic scenarios of how different stressors can simultaneously impact bees. As discussed previously, specific foci should incorporate solitary and wild species, and particularly in field settings to maximize reality.

Initiatives to support bees have been facilitated into agricultural and urban settings over the last decade. However, most of these efforts focus on a handful of common species such as honey bees, instead of rarer species that may be of conservation concern. Moreover, while most restoration projects provide wildflower plantings, they only use a few key plant species that primarily serve the needs of managed bee species. Therefore, the stressors imposed on several species of wild bees resulting from shortages in the specific floral resources or nesting materials necessary for their survival are often ignored. Additionally, while the adoption of pollinator gardens has been widely accepted, they are often implemented and designed without specific conservation objectives. Plus, the goals of conservationists are often not aligned with the desires of commercial agricultural operators or urban personnel. Therefore, future pollinator initiatives should be carefully planned in advance in manners that address the needs of specific bee species of conservation concern within their geographical and ecological area. Surveys of public perception of bees reveal that while most people are enthusiastic about bee health and conservation, their knowledge of basic bee biology and species diversity is largely lacking. Extension and outreach have made some strides to educate agricultural growers and home owners on IPM practices that promote bee health. However, as indicated by these studies, extension programs must improve their efforts to educate the general public on the importance of bees and engage community members in citizen science projects that promote bees and the resources that are necessary for their survival.

Future research should continue to explore effective methods for incorporating floral diversity into agricultural ecosystems, providing bees with ample food resources while enabling growers to still maximize yield. Moreover, if increased yield can be demonstrated through the incorporation of a greater diversity of bees and more diverse habitats surrounding agricultural areas, then grower sentiment toward protecting bees will be enhanced. Increased understanding of major bee disease vectors such as *Varroa* and associated control tactics that do not secondarily harm bees are needed. Additionally, efforts should be undertaken to investigate diseases of less studied native bee species, given recent data suggesting their integral role in crop pollination. The development of target pesticides designed to bind to cell receptors of specific pests while bypassing non-targets including bees is a future endeavor that will hopefully be achieved through molecular and genomic techniques. Assessing non-*Apis* bee pesticide exposure for specific species of interest in methods that examine exposure thorough ecological interactions with their environment are also needed. This will build on another current need for more work on improved timing methods such as spraying before bloom or at times when bees are not present, and on the usage of less toxic chemistries, such as those from a biological origin. Deeper knowledge regarding additive or synergistic impacts on bees resulting from exposure to multiple stressors at a given time will aid in devising improved pollinator protection plans that appropriately address mitigating situations that expose bees to multiple stressors [41]. Further initiatives engaging entire communities in actions to conserve bees (and particularly native species) such as planting native plants and mandating the efficient use of land to promote bee health are also timely. Examples including the projects undertaken in Amsterdam and programs supported by conservation non-profits should be implemented on a wider scale. Their objectives should be incorporated in agricultural settings by promoting positive interaction between growers and beekeepers, and into urban settings by promoting conservation measures in new construction. Educating youth on the importance of bees and their vitality to food production and ecosystem function is the most important.
